# Bacterial Diversity in Traditional Doogh in Comparison to Industrial Doogh

**DOI:** 10.1007/s00284-017-1392-x

**Published:** 2017-11-21

**Authors:** Hamid Reza Sayevand, Farzaneh Bakhtiary, Angelika Pointner, Marlene Remely, Berit Hippe, Hedayat Hosseini, Alexander Haslberger

**Affiliations:** 1 Department of Nutritional Sciences, University of Vienna, Althanstrasse 14, UZAII;2D541, 1090 Vienna, Austria; 2 Faculty of Nutrition Sciences & Food Technology, National Nutrition & Food Technology Research Institute, Shahid Beheshti University of Medical Sciences, Tehran, Iran

## Abstract

Forty-four samples of traditional Doogh and yoghurt were collected from 13 regions of 4 provinces in west of Iran (13 area) and analyzed using molecular methods including PCR, denaturing gradient gel electrophoresis (DGGE) of 16S rDNA, and sequencing. Moreover, collected samples as well as samples from industrially Doogh were analyzed with quantitative real-time PCR (RT-PCR). Analyzed 16S rRNA gene sequences of Doogh samples could be allocated to the presence of *Lactobacillus* spp. The typical yoghurt starter culture bacteria included four different *Lactobacillus* species with possible probiotic properties, *L. acidophilus, L. helveticus, L. kefiranofaciens*, and *L. amylovorus*. DGGE of traditional Doogh and yoghurt and RT-PCR of traditional Doogh and yoghurt and also industrial Doogh samples demonstrated that traditional Doogh and yoghurt show a higher abundance of total bacteria and lactobacilli and a higher bacterial diversity, respectively. Considering diversity and higher probiotic bacteria content in traditional Doogh, consumers’ healthiness in tribes and villages could be promoted with these indigenous products.

## Introduction

**Fig. 1:**
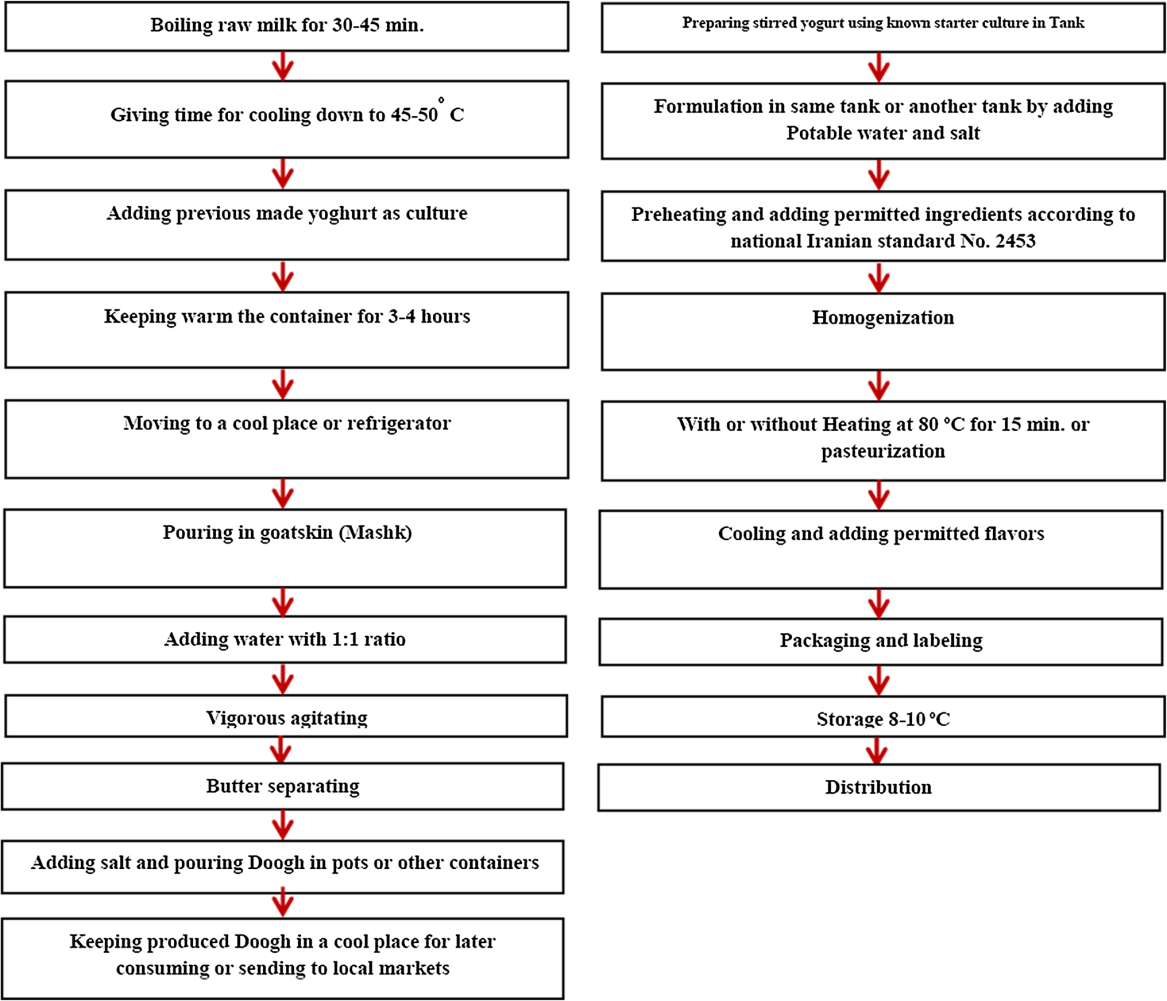
Comparing industrial (left) and traditional Doogh (right) production

Doogh is a fermented milk drink, obtained by dilution of yoghurt with drinking water, addition of salt, followed by heat treatment. However, this definition applies to the production of industrial Doogh with known starter cultures, normally *Streptococcus thermophilus* and *Lactobacillus delbrueckii* subsp. *bulgaricus* [2, 39]. Probiotic industrial Doogh uses specific commercial probiotic strains and is available on the market. Traditional Doogh is buttermilk with specific organoleptic properties and health benefits (Fig. 1), occurring as byproduct of butter production from yoghurt in goatskin or musk bags (Mashk). In traditional Doogh preparation, unknown starter cultures with unidentified characteristics for health and quality (e.g., texture, flavor, and appearance) are used. Additional microorganisms emerge from applied water, Mashk, used equipment etc., further contributing to traditional Doogh’s microbiota composition.

Doogh is common in many parts of Iran and is also popular in Middle East [37]. Due to its acclaimed health benefits it is highly consumed and is a source of probiotic bacteria [16]. Microbial composition of traditional Doogh may vary locally. As yoghurt is the main ingredient of Doogh, it would be expected that the microbial quality of Doogh and yoghurt are similar [2, 31].

In industrial Doogh the initial raw milk, used starter cultures, as well as secondary contaminations like yeast, fungi, and bacteria via equipment, air, ventilation, and packaging material have effects on the product quality and its shelf life [2, 8, 23, 33]. Kefir type drinks and Doogh are produced in the same hygienic conditions in Iranian dairy industry, which may indicate similar bacterial cultures from secondary contamination. Hosseini et al. identified *B. cereus* in industrially produced Iranian Kefir drink [13].

A few studies have been done on comparisons between industrial and traditional Doogh. A study by Jamalifar et al. [17] showed that traditional Doogh samples were able to decrease total *Escherichia coli* O157:H7 to < 10 cfu/ml in a shorter time than industrial and probiotic ones. This suggests that traditional Doogh, but also industrial Doogh to a lesser extent, can both eliminate the risk of bacterial food contamination. This approach is further supported by surveys investigating the presence of *Listeria monocytogenes* in traditionally produced dairy products like Doogh and yoghurt samples, where no listeria could be detected [21, 24, 27, 32].

Along conventional methods like culturing and biochemical tests to study microbiota in Iranian dairy products, molecular methods are applied increasingly [18]. Culture independent methods first, were used for studying ecological aspects of fermented foods but can also be utilized for analysis of microbial spoilage and contaminations [5]. PCR combined with DGGE of amplified 16S rDNA for bacteria and 26S or 18S rDNA for yeasts, respectively, followed by sequencing of the most intense bands, allows to display the most dominant microorganisms in the product [38].

The objective of our study was to analyze the diversity of traditional Doogh microbiota using 16S rDNA PCR–DGGE method and clone library analysis. Genotypic data were supplemented with quantitative real-time PCR (qPCR) analysis comprising comparisons of total bacteria and lactic acid bacteria of traditional Doogh with industrial heat-treated Doogh. Furthermore, we evaluated the traditionally produced yoghurt as source of traditional Doogh microorganisms.

## Materials and Methods

### Samples

Doogh and yoghurt samples were collected from 13 regions in provinces Hamadan, Kohgiloye–Boyerahmad, Chaharmahal–Bakhtiary, and Isfahan including 33 sampling points totally. Samples were transferred into 50-ml sterile falcon tubes directly from the skin bags or the stored batches of the resident families. Sealed samples were stored cool in iceboxes until shipment to the laboratory for analysis the next day.

### Extraction of Bacterial DNA from Doogh and Yoghurt Samples

DNA was extracted by an initial centrifugation (13,000×*g*), followed by lysis and bead beating (30 s, with one intervention minute on ice) of 500 mg of precipitation pellet per sample. Further steps were conducted using the stool mini kit DNA spin kit (Qiagen, USA) following the manufacturer’s protocol. DNA yields and quality were analyzed by Picodrop photometer measurement and gel electrophoresis. Extracted DNA was immediately stored at − 20 °C [11].

### PCR/DGGE

The 16S small subunit ribosomal RNA genes were amplified using the primer set 341f-GC and 518r (Table 1) with a ready-to-use GoTaq^®^ Green Master Mix (Promega, USA). Primer concentration in the reaction volume was 0.5 pM. DGGE gels were prepared as described before by Bakhtiary et al. [3], with a linear gradient of 25–65% using a peristaltic pump and run on 175 V for 6 h as described by Remely et al. and Hosseini et al. [13, 29].

**Table 1:** Primers used for 16S rRNA-based analysis of bacterial communities of Doogh

Target taxon	Primer fragment	Fragment length	Conc. (pmol/µl)	Cycle	Reference
All bacteria	341f-GC 5′-CCT ACG GGA GGC AGC AG-3′	277	10	30	[12, 39]
	518r 5′-ATT ACC GCG GCT GCT GG-3′				
	Uneu F:ACTCCTACGGGAGGCAG	468	10	40	[12, 28]
	Uneu R:GACTACCAGGGTATCTAATCC				
LAB	F: AGCAGTSGGGAATCTTCCA	352–700	4	40	[12, 28]
	R: ATTYCACCGCTACACATG				
TaqMan probe	(FAM)-TAT TAG TTC CTT				[39]
	CAT C-(BHQ-1)				

### Clone Libraries

By means of DGGE gel evaluation, six samples with the largest number of bands on the gel were selected and the PCR products were inserted into a p-GEM Easy Vector system (Promega, USA) following the instructions of the manufacturer. Finally, 60 clones were randomly picked per clone library in TE buffer [30]. The selected clones were amplified with T7: 5′-TAATACGACTCACTATAGGG-′3 and Sp6: 5′-GATTTAGGTGACACTATAG-′3 Promoter Primers (Promega, USA) and then fragment size checked on 2% agarose gel. An additional DGGE analysis with the primers set mentioned in “**PCR/DGGE**” section for selecting clones for sequencing was done. PCR products were then purified using QIAquick PCR Purification Kit (QIAGEN, USA) according to the manufacturer’s instruction and sent for Sanger sequencing as described before by [13, 40].

### 16S rRNA Gene Sequence Analysis

For the purpose of removing parts of the vector, all sequences were corrected using the function “trim vector” in Codon Code Aligner (Codon Code Corporation). Nucleotide sequences were analyzed taxonomically with NCBI (http://blast.ncbi.nlm.nih.gov/Blast.cgi) search and compared to sequencing results in RDP 10.32. (http://rdp.cme.msu.edu/seqmatch/seqmatch_intro.jsp). Phylogenic tree was made according to the NCBI and RDP databanks method [39].

### RT-PCR

The abundance of total bacteria as well as lactic acid bacterial groups in extracted DNA from Doogh samples was measured by 16S rDNA genes using TaqMan qPCR and SYBR Green qPCR in a Rotorgene 3000 (Corbett Life Science, Sydney, Australia) with group-specific Uneu forward and revers primers (Table 1) for all bacteria and Lac1 and Lac2 (Table 1) for lactic acid bacteria, respectively. The temperature profile for qPCR consisted of an initial denaturation step of 10 min at 95 °C followed by 40 cycles of 30 s at 55 °C and 50 s at 72 °C for all bacteria and 10 min at 95 °C followed by 40 cycles of 30 s at 55 °C and 50 s at 72 °C for lactic acid bacteria. All samples were applied in duplicates in RT-PCR. Standard preparation from extracted DNA from *L. Shirota* was done according to [28, 39]. The optimal threshold was selected automatically from regression parameters of the standard curve with the highest R^2^ [39]. The means and their variances were calculated for copies in PCR replicas according to [20]. We used tenfold serial DNA dilutions of *L. Shirota* as well as a mix of strains previously used and described by Pirker et al. [26] to construct standard curves for evaluating PCR reaction efficiency. Further, the copy numbers of these standards were used to calculate DNA copies/µl per sample.

### DGGE Gel Cluster Analysis

DNA fingerprint analysis on the DGGE gels were done by Phoretix 1D Database software free trial (Total Lab Limited 2013, Newcastle, Tyne, England). The software first scans the gel image and after profiling, characterizes individual bands in each profile, with measuring the distance from a defined standard lane. Final clustering results are extracted as a dendrogram. The abundance of bands in each cluster are calculated by counting the bands in each cluster [9].

### Statistical Analysis

All statistical tests were conducted using SPSS 10.0 software. For normality analysis the Shapiro–Wilk’s test was used. Friedman test was applied to investigate differences in band patterns of DGGE bands from different samples. Mann–Whitney test was used to compare the means of diversity between samples. For all comparisons, *P* values of ≤0.05 were considered as significant. All data were shown as mean ± SD [3].

## Results and Discussion

### DGGE and Bacterial Community

**Fig. 2:**
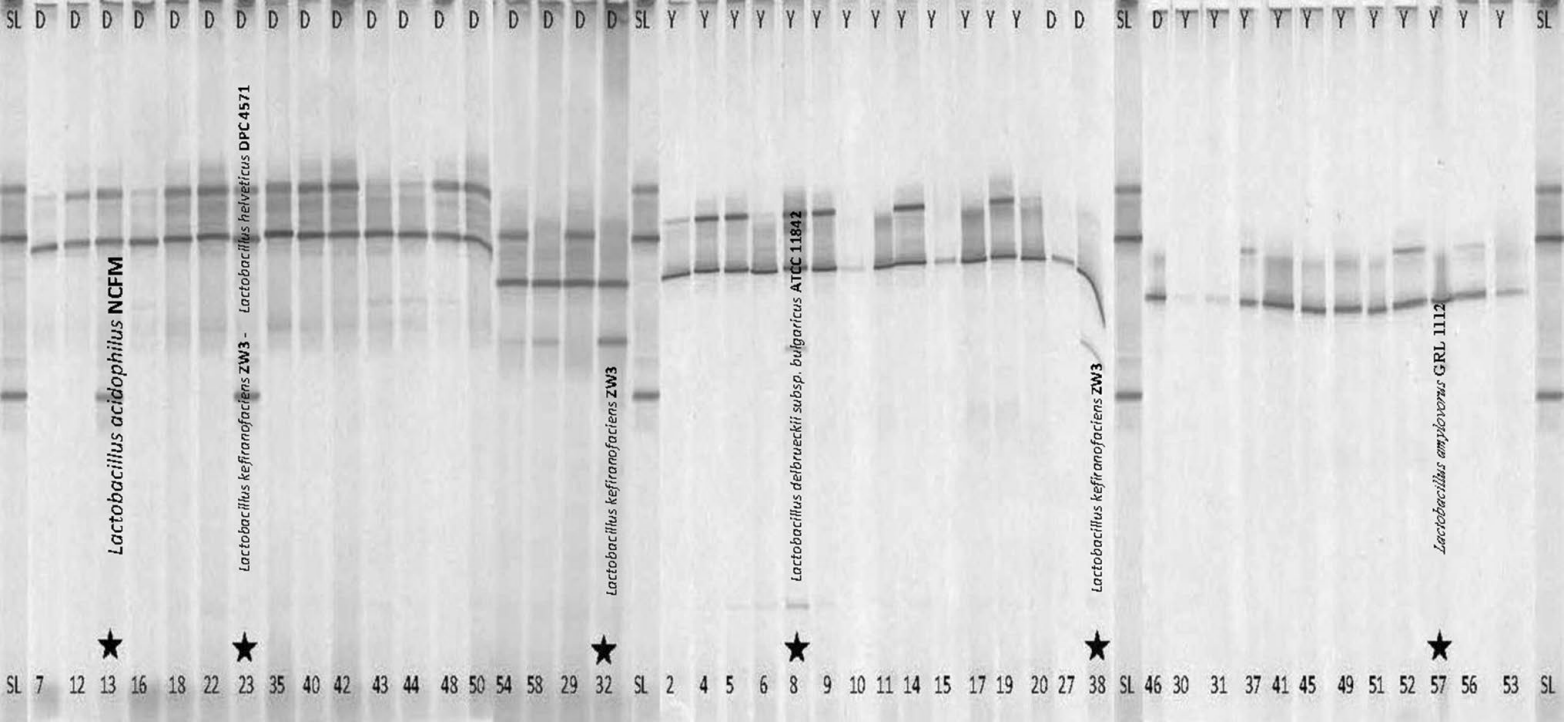
Comparison of DGGE profiles of Doogh and yoghurt samples. Starred lanes are the selected samples for clone library. *D* Doogh and *Y* yoghurt, *SL* standard lane; sample no. 13 repetition

**Fig. 3:**
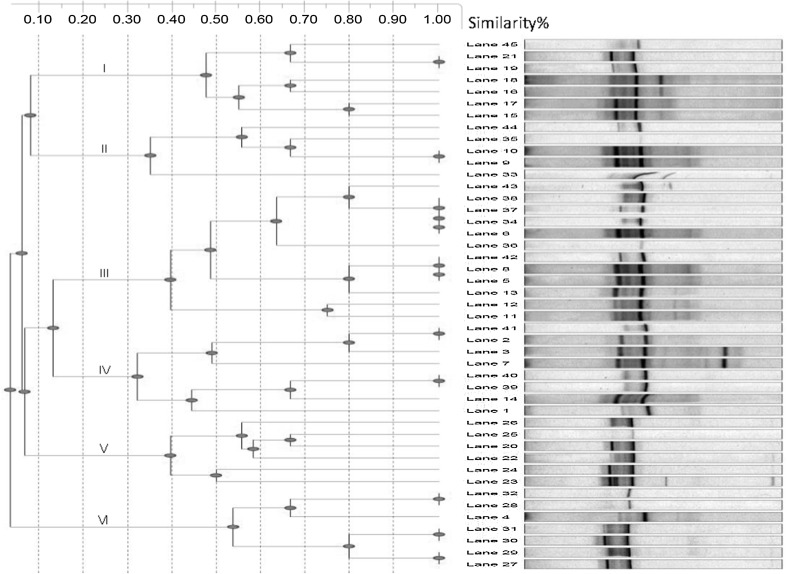
Euclidean-distance dendrogram generated from the DGGE profiles of the 44 samples. The starred lanes are the selected samples for clone library. The scale bar is linkage distance and applies to all dendrogram

**Fig. 4:**
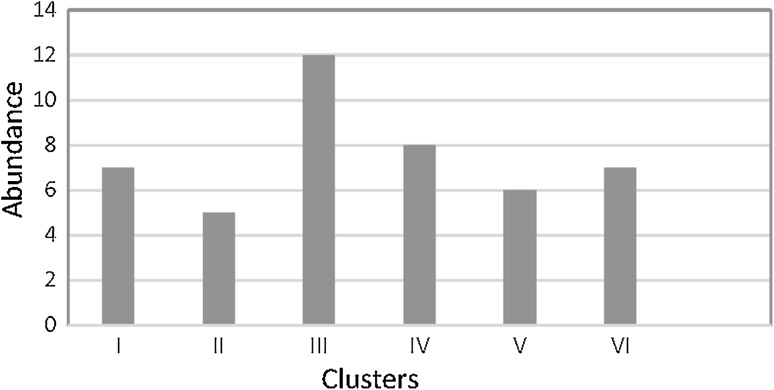
Cluster analysis of Fig. 3 that resulted from counting the number of lanes categorized in each cluster

Aliquots of traditional Doogh and yoghurt samples were extracted and purified. PCR analysis using primers for all bacteria was used to amplify 16S rRNA gene sequences for DGGE, cloning, Sanger sequencing, and phylogenic alignment to study microbial community and potential contamination of traditional Doogh and yoghurt samples. Using primers targeting 16S ribosomal genes of all bacteria helped us to analyze the predominant bacterial strains in traditionally Doogh and yoghurt samples. Comparison of the bacterial DGGE band patterns of traditional Doogh and yoghurt samples showed that many bands (approximately, 212 of 300 bands) (Fig. 2) were similar but the diversity according to number of bands was significantly different (*P* ≤0.05) between traditional Doogh and yoghurt samples. Obviously, similar bands in most samples however show different intensities which can be linked to a higher bacterial abundance detected with RT-PCR (see “Quantification of All Bacteria and Lactic Acid Bacteria in Traditional Doogh and Yoghurt and Industrially Heat Treated Doogh” section). Similarities of DGGE band patterns of Doogh and yoghurt samples indicate that strains might be transferred from yoghurt to Doogh as traditional Doogh is produced from yoghurt. This similarity is probably due to the traditionally used lactic acid bacteria culture in produced yoghurts in all the sampling regions. On average, traditional Doogh samples have a higher diversity compared to yoghurt samples. Bands analysis with DGGE gel dendrogram with cluster analysis revealed six bacterial clusters (Fig. 3 **)**. Furthermore, cluster No. 3 showed a higher abundance (Fig. 4) than the others. This cluster includes samples from five different sampling points (Samples no. 18, 35, 43, 44, 48, 41, 53). Except for sample number 18, members of this cluster are geographically close to each other. Cluster no. 2 includes samples from just one geographic area displaying possible similarities between bacterial communities within an area. In clusters number 1, 4, 5, and 6 at least two geographic areas can be characterized. In total, PCR–DGGE shows the most abundant members in the bacterial community [38]. However, cluster analysis can differentiate between groups but does not characterize the relevance of each operational taxonomic unit, but rather indicates differences [9].

### Clone Libraries and Sequencing

Extracted DNA from samples with higher diversity on DGGE gel showing specific additional bands (Fig. 2) were selected for constructing a clone library (Samples no. 13, 8, 57, 23, 32, 38) and then subjected to Sanger sequencing. By 16S rDNA sequence database searching, six specific clones showed sequence similarities to the origin strain higher than 97%. (Table 2). Some strains of the most abundant detected bacteria groups in samples are *L. acidophilus* NCFM, *L. delbrueckii* subsp. *bulg*aricus ATCC 11842, *L. helveticus* DPC 4571, *L. kefiranofaciens* ZW3 with 20.34% occurrence, and *L.amylovorus* GRL 1112 with 18.64% occurrence (Table 2).

**Table 2:** Randomly picked bacterial clones obtained from traditionally Doogh and yoghurt samples with primer combinations amplifying predominant bacteria

Product	Clone origin sample	Description	Similarity (%)	Accession number	% of randomly picked bacterial clones in a library
Doogh	13	*Lactobacillus acidophilus* NCFM	98	NC_006814.3	20.33
Yoghurt	8	*Lactobacillus delbrueckii* subsp. *bulgaricus* ATCC 11842	99	NC_008054.1	20.34
Yoghurt	57	*Lactobacillus amylovorus* GRL 1112	99	NC_008530.1	18.64
Doogh	23	*Lactobacillus helveticus* DPC 4571	98	NC_010080.1	20.33
Doogh	23, 32,38	*Lactobacillus kefiranofaciens* ZW3	98	NC_015602.1	20.34

### Quantification of All Bacteria and Lactic Acid Bacteria in Traditional Doogh and Yoghurt and Industrially Heat-Treated Doogh

**Fig. 5:**
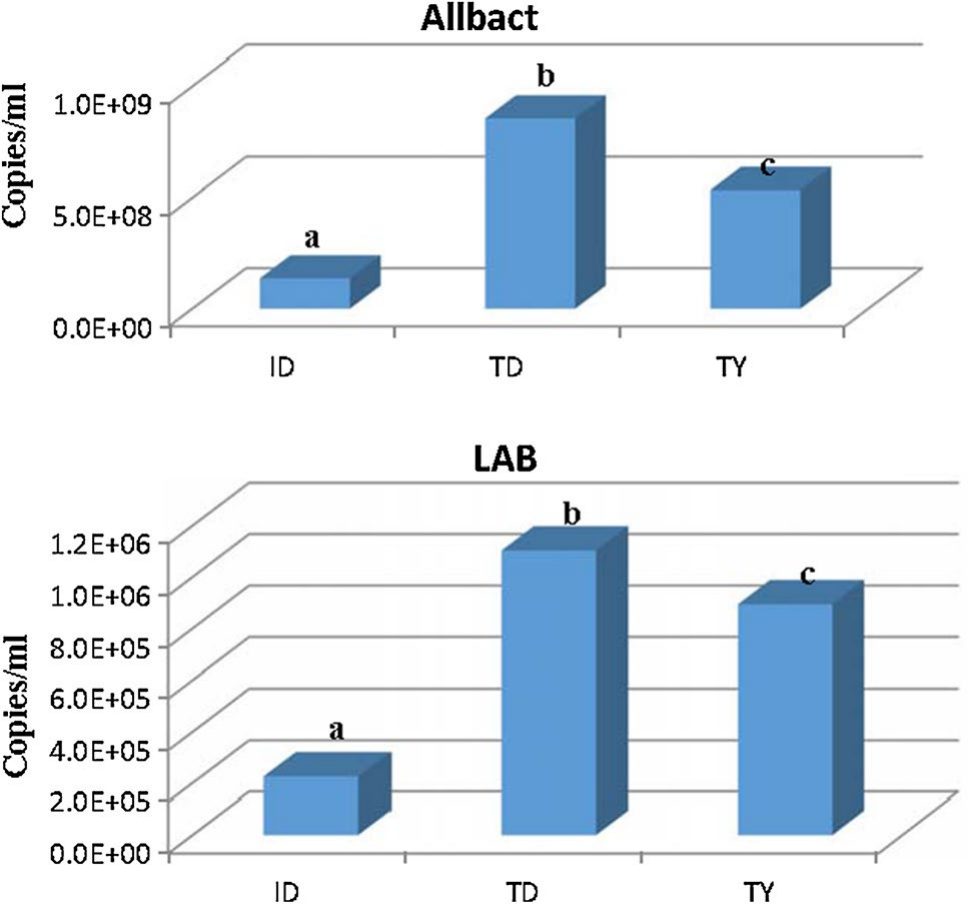
RT-PCR analysis average comparisons copies/ml 16s rDNA region of all bacteria and lactic acid bacteria in extracted DNA from traditional Doogh (TD), traditional yoghurt (TY), and industrial heat-treated Doogh (ID) samples

All bacteria and lactic acid bacteria community in extracted DNA from traditional Doogh and yoghurt and also industrial Doogh samples were quantified with a group-specific SYBR Green and TaqMan RT-PCR assay using standard curve. Comparisons of 16S rRNA copies per milliliter of samples can be seen in Fig. 5. Obviously, in all three analyzed products, the abundance of all bacteria was higher than lactic acid bacteria. Traditional Doogh showed significant (*P* ≤0.05) higher copies of all bacteria as well as lactic acid bacteria compared with the other two products (Fig. 5). An explanation could be that on the one hand the production of Doogh in goat skin bags increases the bacterial content and on the other hand secondary contaminations from used equipment, human, and environment are possible. All of the industrial Doogh samples used in this study were heat treated (pasteurized) so the RT-PCR results on all bacteria and lactic acid bacterial content of industrial Doogh mostly are due to secondary contaminations in industrial Doogh production lines. Most of the contamination of industrial Doogh including nonstarter lactic acid bacteria is because of poor hygienic conditions in dairy factories [2].

## Health Consequences

The investigated bacteria in cloning and sequencing have potential beneficial characteristics for human health. *L. acidophilus* is a homofermentative species, which ferments sugars to lactic acid with optimal growth around 37 °C. *L. acidophilus* naturally can be found in the human and animal gastrointestinal tract and mouth. Some strains (e.g., *L. acidophilus* NCFM) may have probiotic characteristics and they are already commercially used in dairy products [1]. *L. kefiranofaciens* spp. is mostly found in kefir and kefir grains and exhibits exopolysaccharide (EPS) production ability [10, 36]. It is assumed that the produced EPS by this strain and other LAB strains have strong health effects. Positive prebiotic properties on tumors and ulcer as well as an immune stimulating and cholesterol-decreasing effect have been proved [35]. Studies showed that some *Lactobacillus amylovorus* strains isolated from pig fecal had a high tolerance to low pH and bile salts, with antimicrobial properties by bacteriocins and lactic acid production [7, 19]. Omar et al. suggested that consuming *L. amylovorus* as a probiotic yoghurt has the capability to induce total body fat loss by decreasing gut microbial abundance of clostridia [25]. *L. delbrueckii* subsp. *bulgaricus* is the most widely used strain in yoghurt production all over the world. *L. delbrueckii* subsp. *bulgaricus* (strain ATCC 11842/DSM 20081) was originally isolated from Bulgarian yoghurt in 1919 [34]. This strain can tolerate the human gastrointestinal transit conditions with potential probiotic effects [22]. *L. helveticus* spp. was first isolated from Swiss cheese and is used in starter cultures for cheese production with rapid autolysis, reduced bitterness, and increasing flavor characteristics. The genome of this cheese culture is close to health-promoting Lactobacillus strains. Generally, some strains of *L. helveticus* spp. have health effects by proteolytic production of antihypertensive peptides from milk during fermentation [4]. The resulting diversity and community from DGGE and RT-PCR of traditional Doogh and yoghurt samples specify them as indigenous fermented milk products with complex microbiota. The selected samples with unusual bands on DGGE led to at least three possible probiotic lactic acid bacteria strains. As a result, the nature of these products with the proper microbiota possibly has a protective killing effect on most of the existing food pathogens or other contaminants in their matrix and thus display potential health benefits of these traditional product. Industrial Doogh, especially heat-treated types, as they are found in industrial standard starter cultures [6, 14, 15], does not exhibit mixed community of lactic acid bacteria. As a consequence, these products do not share the same health features as traditional Doogh and moreover, may show problems with quality and shelf life. Further studies are needed to characterize all the health aspects of traditional Doogh and yoghurt with consideration of the history of their consumption. There are no studies available about health consequences of traditional Doogh in the areas with high consumption of traditional Doogh and yoghurt. Certainly, our results indicate that traditional Doogh and yoghurt are an exciting source for new effective strains with specific health consequences and furthermore, show potential positive properties in the production process regarding stability of products as well as possible inhibitory activities against contaminants.
